# Injectable Tumoricidal Neural Stem Cell-Laden Hydrogel for Treatment of Glioblastoma Multiforme—An In Vivo Safety, Persistence, and Efficacy Study

**DOI:** 10.3390/pharmaceutics17010003

**Published:** 2024-12-24

**Authors:** Jasmine L. King, Alain Valdivia, Shawn D. Hingtgen, S. Rahima Benhabbour

**Affiliations:** 1 Joint Department of Biomedical Engineering, The University of North Carolina at Chapel Hill and North Carolina State University, Chapel Hill, NC 27599, USA; jasmine_king@med.unc.edu; 2 Division of Pharmacoengineering and Molecular Pharmaceutics, UNC Eshelman School of Pharmacy, University of North Carolina at Chapel Hill, Chapel Hill, NC 27599, USA; alain07@email.unc.edu (A.V.); hingtgen@email.unc.edu (S.D.H.)

**Keywords:** neural stem cells, stem cell engineering, stem cell delivery, hydrogels, injectable hydrogels, hydrogel delivery systems, post-surgical glioblastoma multiforme, glioblastoma multiforme

## Abstract

Background/Objectives: Glioblastoma multiforme (GBM) is the most common high-grade primary brain cancer in adults. Despite efforts to advance treatment, GBM remains treatment resistant and inevitably progresses after first-line therapy. Induced neural stem cell (iNSC) therapy is a promising, personalized cell therapy approach that has been explored to circumvent challenges associated with the current GBM treatment. Methods: Herein, we developed a chitosan-based (CS) injectable, biodegradable, in situ forming thermo-responsive hydrogel as a cell delivery vehicle for the treatment of GBM. Tumoricidal neural stem cells were encapsulated in the injectable CS hydrogel as stem cell therapy for treatment of post-surgical GBM. In this report, we investigated the safety of the injectable CS hydrogel in an immune-competent mouse model. Furthermore, we evaluated the persistence and efficacy of iNSC-laden CS hydrogels in a post-surgical GBM mouse model. Results: The injectable CS hydrogel was well tolerated in mice with no signs of chronic local inflammation. Induced neural stem cells (iNSCs) persisted in the CS hydrogels for over 196 days in comparison to 21 days for iNSCs (cell injection) only. GBM recurrence was significantly slower in mice treated with iNSC-laden CS hydrogels with a 50% increase in overall median survival in comparison to iNSCs (cell injection) only. Conclusions: Collectively, we demonstrated the ability to encapsulate, retain, and deliver iNSCs in an injectable CS hydrogel that is well tolerated with better survival rates than iNSCs alone.

## 1. Introduction

Glioblastoma multiforme (GBM) is the most complex, treatment-resistant form of brain cancer and accounts for 49% of all primary malignant brain cancers [1]. The standard approach for treatment of GBM consists of maximal surgical resection followed by concomitant chemoradiation [2,3]. The median time for recurrence is estimated to be between 6 and 9 months and average length of survival for patients diagnosed with GBM is approximately 12–15 months after initial diagnosis [4,5]. Currently, there is no clinical consensus for treatment after recurrence and options are often limited. Despite decades of research, new therapies are warranted to improve quality of life, reduce mortality, and improve clinical outcomes for GBM patients. Over the past 20 years, neural stem cells (NSCs) have been investigated as a novel modality for the treatment of brain cancer. To date, there have been several clinical trials investigating the intracerebral administration of allogeneic NSCs engineered to deliver enzyme-mediated prodrugs or oncolytic viruses for newly diagnosed or recurrent GBM [6]. Although results from these clinical trials demonstrated the safety, feasibility, and non-tumorgenicity of allogeneic NSCs, careful long-term monitoring may be warranted because of potential immune-related responses and immune rejection associated with its use. Autologous, patient-derived NSCs can pose as an advantageous therapeutic strategy to circumvent these challenges. However, isolating a pure population of NSCs from adult patients requires invasive procedures and significant safety concerns [7].

Hingtgen and colleagues have discovered a novel reprogramming strategy that consists of isolating differentiated somatic cells and converting them into induced neural stem cells (iNSCs), a process termed transdifferentiation (TD) [8,9,10]. This process uses a tetracycline-inducible, single SOX2 transcription factor that shortens the transduction process to 5 days. Studies have shown a high transduction efficiency and the ability to maintain their expression of NSC markers over time [9]. Additionally, preclinical studies have shown that iNSCs possess unique self-renewal properties, can be engineered to secrete anticancer proteins such as secretable proapoptotic protein tumor necrosis factor-α-related apoptosis-inducing ligand (sTRAIL; sTR), and are inherently tumoritropic [8,9,10,11]. Although iNSCs have been shown to effectively target GBM cells, their poor residence time within the cranial cavity reduces overall efficacy and survival outcomes [8,9]. Therefore, the intra-cavity delivery, retention, and persistence of iNSCs need significant improvement.

Biomaterials can be designed and engineered to support the sustained delivery of therapeutic iNSCs and enhance the treatment of GBM. Currently, there are no biomaterials marketed or in clinical development that effectively improve iNSC persistence and GBM treatment outcomes. This is mainly attributed to variable efficacy outcomes reported with biomaterial scaffolds for the delivery of iNSCs due to limited cell seeding/density within the scaffold and/or poor cell homogeneity within the scaffold, which can lead to inconsistent dosing in the surgical cavity [12,13]. Other potential challenges include the need for repeat invasive surgery to remove the biomaterial if it is not biodegradable in nature. Hydrogels have been widely used as supportive carrier systems to deliver therapeutic cells for tissue engineering, cancer, and other biomedical applications [14]. Hydrogels can be engineered via 3D bioprinting or as injectables using chemical or physical crosslinking strategies. Particularly, injectable, in situ forming, thermo-sensitive hydrogels undergo fast gelation upon temperature changes. In recent years, there have been numerous studies exploring in situ and/or thermo-responsive hydrogels for the delivery of stem cell therapeutics [15,16,17,18]. These studies have included investigative approaches aimed at delivering mesenchymal and neural stem cells engineered to express enzymes and secrete proteins, such as thymidine kinase and TRAIL [19]. However, there is only one study that explored the delivery of TRAIL-secreting human iNSCs in three polymer matrices: a fibrin only product—TISSEL, gelatin-based matrix—Gelfoam, and a hemostatic matrix—FLOSEAL [20]. In this study, FLOSEAL was the only matrix that extended persistence of iNSCs beyond 3 weeks. However, there were no significant differences in survival with the use of FLOSEAL in comparison to direct injection of iNSCs in U87-MG-bearing animals. Therefore, there is a clinical need to significantly improve survival outcomes in U87-MG xenograft models.

Chitosan-based hydrogels have been extensively explored as a suitable cell delivery system for tissue engineering and cancer [21,22]. Chitosan is thermo-sensitive in nature and possesses biodegradable, biocompatible, antimicrobial, and non-immunogenic properties that make it an ideal candidate for a drug/cell delivery system for the treatment of cancer [23,24,25,26]. Additionally, chitosan can be (1) formulated as an injectable, in situ forming hydrogel that exhibits shear-thinning behavior with tunable mechanical properties to mimic host tissue and control the release of drugs/cells at the target site. To our knowledge, this is the first in vivo study exploring the safety, efficacy, and survival rate of an injectable, in situ forming, CS-based hydrogel scaffold to “house” and deliver tumoricidal iNSCs for post-surgical U87-MG GBM xenografts. In this study, we demonstrate that CS hydrogels significantly improve the persistence of iNSCs, extend the median overall survival in U87-MG xenografts, and are notably safe following implantation. These results demonstrate the therapeutic durability, safety, and promise of this technology for post-surgical GBM. Furthermore, it will begin to define new, adjuvant therapeutic approaches for primary resectable GBM.

## 2. Materials and Methods

### 2.1. Materials

Chitosan (CS) powder (85% deacetylated, MW: 310–350 kDa, 200–800 cP, and 3 wt% in 0.1 M acetic acid), β-glycerophosphate (BGP), and hydroxyethyl cellulose (HEC, MW: 90,000 Da) were purchased from Sigma-Aldrich (St. Louis, MO, USA). Luer-lock (1 mL) syringes were purchased from Becton and Dickinson (Franklin Lakes, NJ, USA). Luer-lock connectors were purchased from Baxter (Deerfield, IL, USA). BD PrecisionGlide 18 G × 0.5” hypodermic blunt tip needles were purchased from Becton and Dickinson (Franklin Lakes, NJ, USA). Falcon™ Cell Strainers (100 µm mesh size) were purchased from Fisher Scientific (Pittsburgh, PA, USA). D-luciferin potassium salt bioluminescent substrate was purchased from ThermoFisher Scientific (Waltham, MA, USA). Standard cell culture media was prepared using Dulbecco’s Modified Eagle Medium (DMEM; Gibco; Detroit, MI, USA) supplemented with 10% fetal bovine serum (FBS; Gibco; Detroit, MI, USA), 1% penicillin/streptomycin (Gibco; Detroit, MI, USA), and 4 mM L-glutamine. Dulbecco’s Phosphate Buffered Saline (DPBS; Gibco; Detroit, MI, USA) was purchased from the University of North Carolina Tissue Culture Facility (Chapel Hill, NC, USA). STEMdiff™ Neural Induction Media, Accutase™ Cell Detachment Solution, and doxycycline powder were purchased from STEMCELL Technologies (Cambridge, MA, USA).

### 2.2. Cell Lines and Lentiviral Vectors

U87-MG cells were purchased from the American Type Culture Collection (ATCC; Manassas, VA, USA). Normal human fibroblast (NHF-1) was gifted from W. Kaufmann at the University of North Carolina School of Medicine (Chapel Hill, NC, USA). Lentiviral vectors were gifted from the Duke University Viral Vector Cores (Durham, NC, USA): (1) reverse tetracycline-controlled activator (rtTA), (2) doxycycline inducible SOX2 (SOX2), (3) mCherry-Firefly Luciferase (mCh-FLuc), (4) green fluorescent protein-Firefly Luciferase (GFP-FLuc), and (5) GFP fused with a secretable TRAIL variant (GFP-sTRAIL; GFP-sTR).

### 2.3. Generation of iNSCs for Survival and Therapy

iNSCs were generated from an established protocol [9]. In brief, 2 × 10^6^ infected NHF-1 cells were plated in a T-175 and cultured in a standard cell culture media (day 1). On day 2, the standard cell culture media was aspirated and replaced with a Neural Induction Media containing 2 µg/mL doxycycline (NIM+doxy also known as transdifferentiation (TD) media). Thereafter, the TD media was replaced every other day for 4 days. On day 6, iNSCs were collected using an Accutase Cell Detachment Solution and filtered through a 100 µm cell strainer.

### 2.4. Hydrogel Preparation and Cell Encapsulation

The hydrogels were prepared from an established protocol [25,27]. CS hydrogels were prepared using a conventional luer-lock-to-luer-lock connector method. In brief, 2% (*w*/*v*) chitosan solution (CS; syringe 1) was uniformly mixed with the primary gelling agent, β-glycerophosphate (BGP; syringe 2). The resulting mixture was then mixed with the secondary gelling agent, hydroxyethyl cellulose (HEC; syringe 3), to form the pre-hydrogel mixture. To encapsulate iNSCs, iNSCs were collected and filtered as previously described. The cells were centrifuged at 1000 rpm for 5 min to form a cell pellet. The cell pellet was resuspended in 1X DPBS and combined with the HEC solution for encapsulation into the hydrogel.

### 2.5. In Vivo Models

#### 2.5.1. Stem Cell Retention and Persistence

All animal procedures were approved by the Animal Care and Use Committee at the University of North Carolina at Chapel Hill. Nude mice (6–8 weeks old; Animal Studies Core, University of North Carolina at Chapel Hill) were anesthetized using 2% inhaled isoflurane and immobilized on a stereotactic frame. The surgical site was sterilized with 70% isopropyl alcohol and betadine. A midline incision was made to expose the cranium of the mouse. To mimic a resection cavity after tumor removal, a craniectomy was performed using a high-speed micro drill. At day 7 post craniectomy, the underlying dura was removed and peeled back using surgical forceps and cotton swabs. Next, a 3 × 3 mm region of the cerebral cortex and white matter was removed, at the junction of the parietal and occipital lobes. To determine retention and persistence of iNSCs, iNSCs-GFP-FLuc (2.5 × 10^4^ cells per 5 µL in sterile DPBS; n = 5) were directly injected into the mock resection cavity or were encapsulated in the CS hydrogel (2.5 × 10^4^ cells per 5 µL hydrogel; n = 5) and implanted into the mock resection cavity. The skin was closed using surgical glue and the mice were placed into the recovery chambers following surgical procedures. Serial bioluminescence imaging (BLI) was performed on days 1, 4, 7, and weekly thereafter post-implantation until complete signal loss.

#### 2.5.2. Murine Safety Studies

Female C57BL/6J mice (6–8 weeks old; The Jackson Laboratory, Ban Harbor, ME, USA) were anesthetized using 2% isoflurane and immobilized in the prone position on the stereotactic frame. Prior to the surgical procedure, mice were divided into the following treatment groups: (1) CS hydrogel, (2) resection control, and (3) sham. Hair was removed from the surgical site using an electric clipper. As previously mentioned, the surgical site was sterilized, and a midline incision was made to expose the cranium. Using the methods previously discussed, a mock resection cavity was created and 5 µL of placebo cell free CS hydrogel was implanted in the resection cavity (n = 5). In a subset of mice (n = 3), mock resection cavities were performed to serve as a positive control. Additionally, a sham (no mock resection, healthy mice) group (n = 3) was included in this study to serve as a negative control. To determine the safety profile of the CS hydrogel, local inflammation was assessed on days 3, 7, 30, 60, and 90 post-implantations.

#### 2.5.3. Post-Surgical Anti-GBM Efficacy of iNSC-s-TR-Hydrogel Therapy

Nude mice (6–8 weeks old; Animal Studies Core, University of North Carolina at Chapel Hill) were anesthetized using 2% inhaled isoflurane and immobilized on a stereotactic frame. The surgical site was sterilized with 70% isopropyl alcohol and betadine. A midline incision was made to expose the cranium of the mouse. As previously described, a craniectomy was performed. Using a Hamilton syringe, U87-MG mCh-FLuc (5 × 10^4^ cells per 3 µL in sterile DPBS) was implanted stereotactically in the right frontal lobe of the mice using the following coordinates in reference to the bregma (x-axis: 2 mm; y-axis: 1 mm; z-axis: 1.5 mm) 7 days post craniectomy. After 7 days, the incision was reopened, and the underlying dura was removed in the cranial window to expose the tumor. Using image-guidance, the U87-MG mCh-FLuc tumor was excised using surgical dissection and aspiration under mCherry excitation. Following tumor removal, the iNSCs-GFP-NLuc (n = 5) for treatment group (1), or the iNSCs-GFP-sTR (2.5 × 10^4^ cells per 5 µL in sterile DPBS; n = 10) for treatment group (2) was directly injected into the resection cavity or encapsulated in CS hydrogel (2.5 × 10^4^ cells per 5 µL hydrogel; n = 10) for treatment group (3) and implanted into the surgical cavity. Tumor regrowth was monitored using serial BLI and the animals were tracked to determine survival in each group. All animals were routinely monitored for severe adverse events, weight loss, pain, or signs of infection.

### 2.6. In Vivo Bioluminescence Imaging (BLI)

D-Luciferin was reconstituted in sterile 1X DPBS (15 mg/mL) and administered intra-peritoneally (IP; 150 mg/kg body weight) 15 min before imaging. The IVIS^®^ Spectrum in vivo imaging system was used to non-invasively measure cell activity and monitor disease progression in vivo. Photon emission was determined using the Living Image^®^ software (v. 4.3.1) and expressed as total photon flux (photons/second; p/s).

### 2.7. Tissue Processing

At the study endpoints, mice were anesthetized with an IP injection of Ketamine/DexDomitor in sterile DPBS (15 mL/kg). A thoracotomy was performed, and mice were perfused with 10 mL sterile DPBS followed by 10 mL of 10% formalin. Brain tissue was harvested and stored in 10% normal buffered formalin (NBF) in preparation for histological analysis.

### 2.8. Histological Staining and Analysis

Formalin-fixed brain tissue samples were paraffin embedded and sectioned at 5 µm thickness. Tissue sections were stained with hematoxylin and eosin, and histopathological analysis was performed by the University of North Carolina at Chapel Hill Director of Pathology Core, Board Certified Veterinary Pathologist (R. S. S).

### 2.9. Statistical Analysis

Data were analyzed by performing a two-way ANOVA multiple comparisons test using GraphPad Prism Software v.9.3.1. In this study, data were expressed as the mean ± standard deviation (SD) and statistically significant differences between groups were set at a threshold of *p* < 0.05. Survival times of mice groups were compared using the logrank (Mantel–Cox) test.

## 3. Results

### 3.1. Preparation of Injectable CS Hydrogels Bearing iNSCs

**Figure 1:**
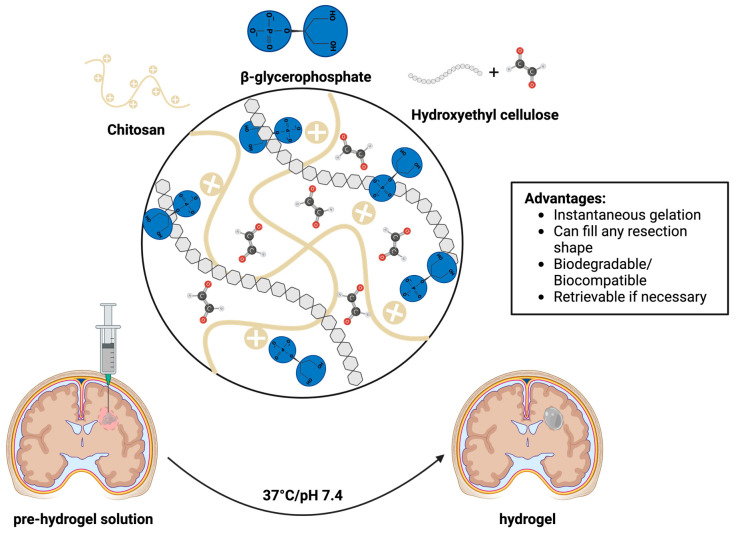
Schematic illustration of the injectable CS hydrogel under physiological conditions.

In previous studies, we have shown that the injectable chitosan (CS)-based hydrogel was suitable for neural stem cell encapsulation [27]. In these studies, we highlighted key advantages of the CS hydrogel delivery system (Figure 1). The CS hydrogel exhibited (1) fast gelation kinetics under physiological conditions and (2) excellent biodegradability and cytocompatibility. In addition to these characteristics, this hydrogel system can (1) fill any resection shape and (2) be retrievable if necessary.

**Figure 2:**
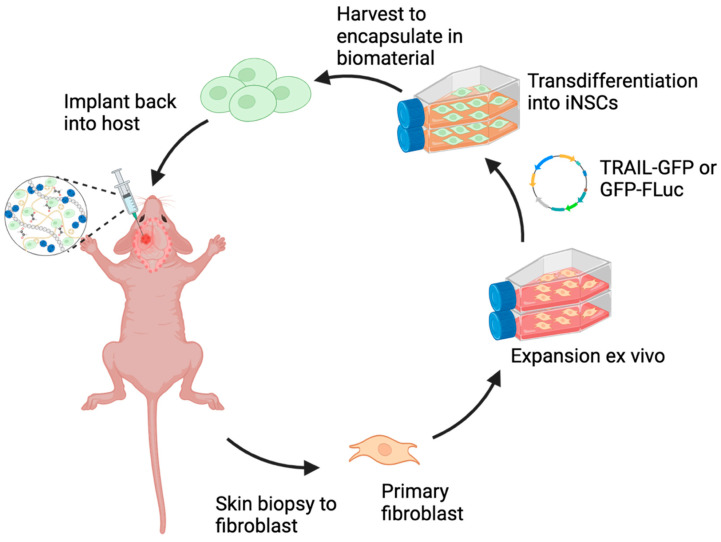
Schematic illustration depicting the transdifferentiation (TD) process of primary fibroblast to induced neural stem cells for implantation.

Based on previous studies, we optimized the hydrogel formulation composition for cell delivery as illustrated in Figure 2. In brief, 2% CS is homogenously mixed with 100 mM of BGP via a luer-lock adapter. The resultant syringe is then mixed with HEC and the cell suspension to prepare a pre-hydrogel mixture. Under physiological conditions (pH 7.4, 37 °C), the pre-hydrogel mixture undergoes a sol-gel transition to form a hydrogel instantaneously [25,27]. The primary mechanism of crosslinking is physical crosslinking via electrostatic interactions and hydrogen bonding. The secondary mechanism of crosslinking is primarily governed by covalent bonding via a Schiff base reaction between the CS backbone and the reactive glyoxal molecules in HEC. The synthesis of the hydrogel was previously characterized via Fourier-transform infrared (FTIR) spectroscopy analysis [25]. FTIR analysis revealed an absorbance at wavenumbers from 3300 to 3400 cm^−1^ which were characteristic peaks of hydrogen bonding [25]. Additionally, the FTIR spectra revealed an absorbance at 2360 cm^−1^ which confirmed the chemical crosslinking between the amine groups within the CS backbone and the glyoxal molecules in HEC [25]. Moreover, the swelling and mechanical properties of the CS hydrogel were previously investigated, and the results demonstrated that the CS hydrogel exhibited fast shrinkage (50%) within 24 h incubation followed by slow shrinkage, reaching ~68% after 42 days incubation in PBS at 37 °C. Results from mechanical testing demonstrated that the CS hydrogels elicited an elastic modulus comparable to human brain tissue, 6.713 kPa vs. 1–100 kPa, respectively [28,29]. Previously, we evaluated the cytocompatibility and killing potential of iNSCs in the CS hydrogel [27]. These studies demonstrated that 5 × 10^6^ cell per mL of hydrogel was the optimal cell density to maintain > 80% cell viability following encapsulation, to efficiently secrete therapeutic levels of TRAIL (>50 ng/mL), and to effectively kill > 50% of the GBM cells in vitro within 72 h [27]. Therefore, the hydrogel formulations illustrated in Table 1 were selected for use in this study to investigate persistence, safety, and efficacy in vivo.

To generate iNSCs, a single SOX2 transcription factor TD method has been established to accelerate cell reprogramming to be consistent with the clinical timeline for GBM care (outlined in Figure 2). Previous studies have characterized and confirmed the conversion of SOX2/NHF-1 to iNSCs using this TD strategy via immunofluorescence staining and genetic analysis [9]. To monitor iNSCs in vivo, NHF-1s were expanded in a culture dish and genetically engineered with optical reporters, GFP, and FLuc. Following the TD process, iNSCs were encapsulated in the pre-hydrogel solution for implantation (Figure 2). As previously mentioned, the CS hydrogel was designed to enhance retention and persistence of iNSCs within the resection cavity and allow cell migration and release of cytotoxic molecules from cells to kill residual GBM tumor foci following surgery and to suppress GBM recurrence.

### 3.2. Enhancing the Retention and Persistence of iNSCs Using an Injectable CS Hydrogel

**Figure 3:**
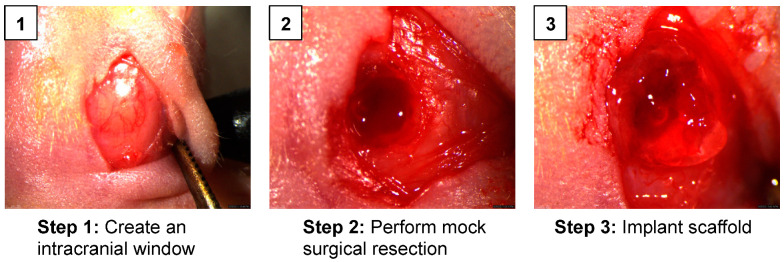
Surgical procedure for in vivo persistence studies. (**1**) In step 1, a surgical incision was created to expose the intact skull, and an intracranial window (craniotomy) was created in the right hemisphere of the parietal skull plate using a microsurgical drill. (**2**) In step 2, using a surgical scope, an aspiration device was used to remove brain tissue to create a mock surgical resection cavity. (**3**) In step 3, a 5 µL of CS hydrogel solution containing 25,000 iNSCs was implanted into the resection cavity. The iNSC-CS hydrogel was given 1–2 min to settle before closing the wound with Vetbond tissue adhesive (3M 1469SB).

**Figure 4:**
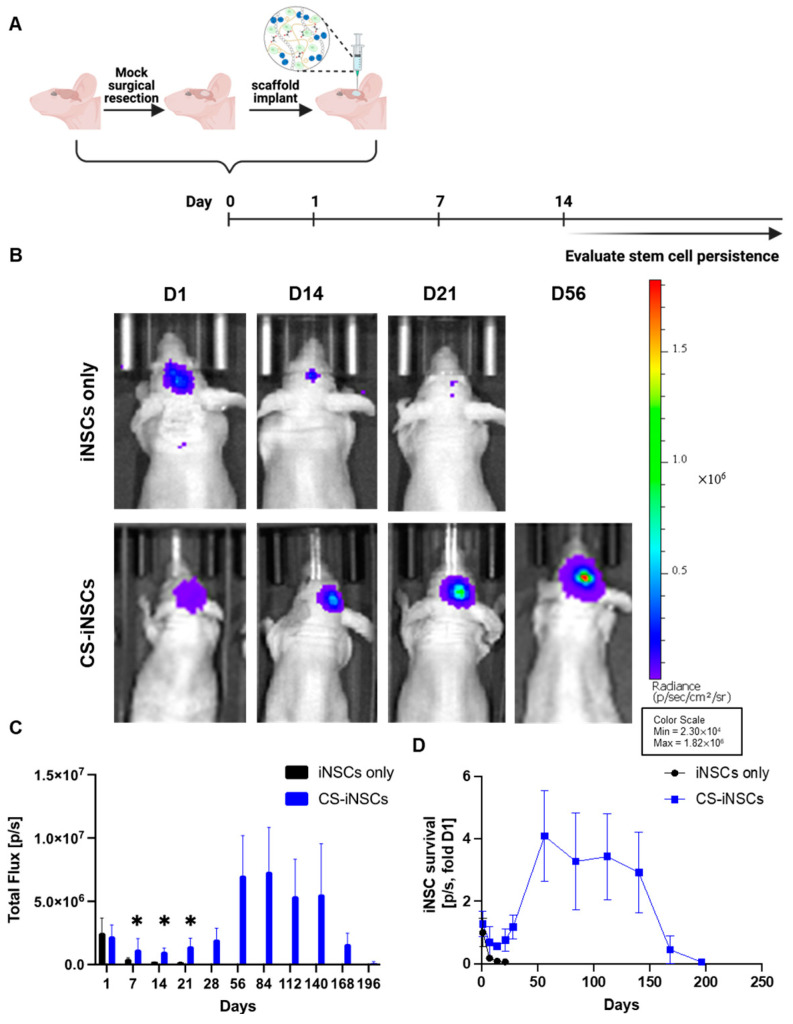
In vivo retention and persistence post-implantation of iNSCs via direct injection or seeded in CS hydrogels. (**A**) In vivo persistence study design. (**B**) Representative BLI images collected up until signal loss for each group. (**C**,**D**) Summary graphs demonstrating the FLuc signal from iNSCs directly injected or encapsulated in CS hydrogel following delivery into the resection cavity. iNSC survival was represented as total FLuc signal from day 1 (* indicates *p* < 0.05 by two-way ANOVA).

Effective stem cell therapies for post-surgical GBM must reside within the resection cavity for a sufficient period of time to exert its anti-tumor activity. To mimic GBM resection clinically, we used an established GBM resection mouse model to assess the retention and persistence of iNSCs in vivo [12,13,19,30]. Mock surgical resections were created using established methods by Hingtgen et al., as demonstrated in Figure 3. iNSCs-GFP-FLuc were encapsulated in the CS hydrogel matrix and implanted within the resection cavity as shown in step 3 of Figure 3 (2.5 × 10^4^ iNSCs per 5 µL injection; n = 5). Alternatively, an equal number of iNSCs-GFP-FLuc in sterile 1X DPBS were directly injected into the resection cavity to simulate cell-free implantations as shown in phase I clinical trials [31]. The FLuc signal was evaluated using BLI 24 h post-implantation and weekly thereafter (Figure 4A). Real-time noninvasive imaging showed that iNSCs directly injected in the resection cavity persisted for approximately 21 days (Figure 4B and Supplementary Figure S1). Conversely, iNSCs encapsulated within the CS hydrogel matrix persisted for 168 days in all animals and up to day 196 in one animal (Supplementary Figure S1). It should be noted that one out of the five animals experienced complications during implantation and was euthanized following the surgical procedure. Additionally, animals 007 and 008 were not imaged on day 196 due to health concerns. These results demonstrated that CS hydrogels significantly increased stem cell persistence in the surgical resection cavity, extending overall persistence by 9.3-fold (Figure 4C,D). Together, these findings suggest that the CS hydrogel significantly delays the clearance of iNSCs from the resection cavity thus improving the durability of iNSCs for anti-GBM therapy.

### 3.3. Assessing the Safety of Injectable CS Hydrogels

**Figure 5:**
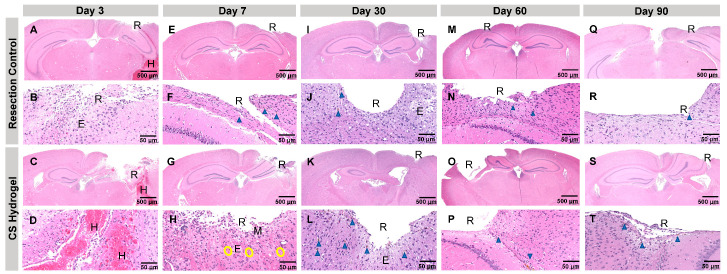
Histology and safety of mice brain tissue following the resection and the CS hydrogel implantation. Histological images of brain tissue on days 3, 7, 30, 60, and 90 following the resection (n = 3/timepoint) and post-implantation of the CS hydrogel (n = 5/timepoint). (**A**,**B**) Resection control histological images at day 3 (**A**) and (**B**) a zoomed-in image of (**A**). (**C**,**D**) CS hydrogel histological images at day 3 (**C**) and (**D**) a zoomed-in image of (**C**). (**E**,**F**) Resection control histological images at day 7 (**E**) and (**F**) a zoomed-in image of (**E**). (**G,H**) CS hydrogel histological images at day 7 (**G**) and (**H**) a zoomed-in image of (**G**). (**I**,**J**) Resection control histological images at day 30 (**I**) and (**J**) a zoomed-in image of (**I**). (**K**,**L**) CS hydrogel histological images at day 30 (**K**) and (**L**) a zoomed-in image of (**K**). (**M**,**N**) Resection control histological images at day 60 (**M**) and (**N**) a zoomed-in image of (**M**). (**O**,**P**) CS hydrogel histological images at day 60 (**O**) and (**P**) a zoomed-in image of (**O**). (**Q**,**R**) Resection control histological images at day 90 (**Q**) and (**R**) a zoomed-in image of (**Q**). (**S**,**T**) CS hydrogel histological images at day 90 (**S**) and (**T**) a zoomed-in image of (**S**). R represents resection site, H represents hemorrhage (trauma-related), E represents edema (trauma-related), yellow circles represent swollen axons (trauma-related), and blue triangles represent pigment-laden macrophages. All scale bars represent 500 µm. All scale bars for zoomed-in images represent 50 µm.

Local tolerability of CS hydrogel was assessed in C57BL/6J immunocompetent mice and compared to resection control mice that only underwent mock surgical resections. (Figure 5). In this study, there were 15 (n = 3/timepoint) untreated control mice that served as the healthy standard control group (Supplementary Figure S2), 15 (n = 3/timepoint) mice that served as the resection control group, and 25 (n = 5/timepoint) CS hydrogel treated mice that served as the experimental treatment group. Brain tissue was harvested, sectioned coronally, and hematoxylin and eosin (H&E) staining of the resection site was performed on days 3, 7, 30, 60, and 90.

Histological staining analysis of the brain tissue demonstrated that the inflammation present at the surgical site was attributed to tissue damage associated with the surgical procedure. Moreover, the most significant findings were all attributed to the surgical procedure (microscopic findings summarized in Supplementary Figure S3). As indicated in Figure 5A–D, hemorrhaging in the parenchyma and lateral ventricles was of minimal to moderate in severity at day 3 in the CS hydrogel group and of minimal severity in the resection control group. Additionally, neuronal necrosis and axon swelling was identified in the tissues adjacent to the surgical resection site. Edema, indicated with E, was identified around the surgical site and was limited to the parenchyma immediately adjacent to the surgical site. Gliosis, also known as the repair response, was also present in the parenchyma immediately adjacent to the surgical site. This finding was minimal in severity and more evident on day 7 in comparison to day 3. Mixed inflammation, fibrin, and minerals were identified in one CS hydrogel treated animal at day 3 and one at day 7. This finding was not evident in the resection control treated animals (Supplementary Figure S4). This finding was more likely related to surgery with bone trauma and local tissue necrosis. At days 30, 60, and 90, no tissue samples had any notable inflammation. Additionally, there was very minimal evidence of injury beyond minimal to mild edema in the region around the resection site.

### 3.4. Tumoricidal iNSC-sTR Therapy for Post-Surgical GBM Treatment in Human GBM Xenografts

**Figure 6:**
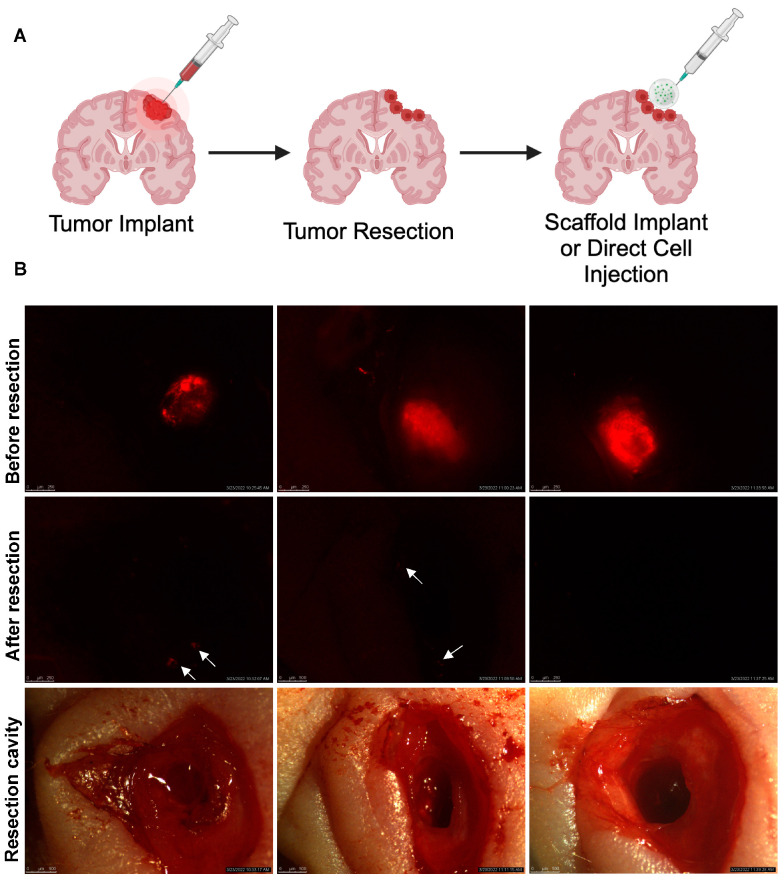
(**A**) Schematic illustration of tumor implantation, resection, and cell injection/implantation in vivo. (**B**) Fluorescent images of U87-MG mCh-FLuc tumors before and after resection and images of resection cavity using an Olympus MVX-10 microscope (1.6× magnification). White arrows represent positive tumor margins following resection.

**Figure 7:**
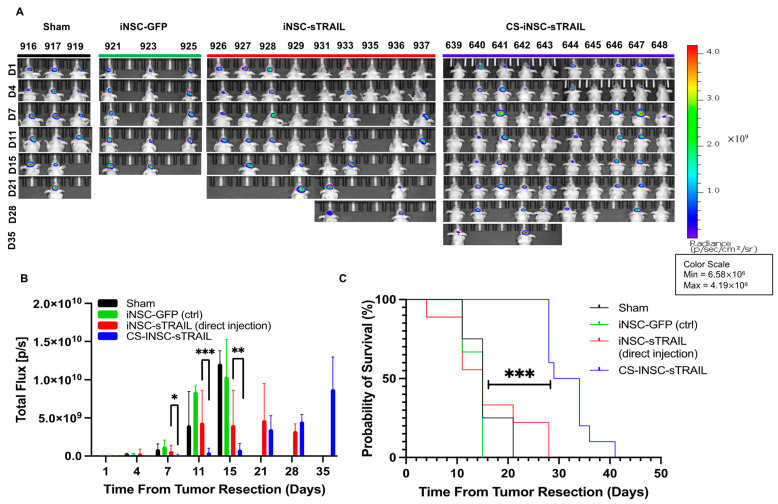
Delivery of tumoricidal iNSCs to inhibit progression of GBM in post-resection model. (**A**) Representative images of serial BLI showing tumor inhibition and regrowth in iNSC-sTR treated versus control-treated animals. (**B**) Summary graph depicting the tumor radiance of U87-MG FLuc overtime following post-resection treatment (* indicates *p* < 0.05; ** *p* < 0.005; *** *p* < 0.001 by ANOVA). (**C**) Kaplan–Meier survival analysis demonstrating the survival of animals receiving iNSC-sTR therapy in comparison to control-treated animals (*** *p* < 0.001 by log-rank test).

Surgery is the initial standard of care approach for the treatment of primary GBM inpatients. To determine the clinical practicality of this combinatory cell-biomaterial technology, a U87-MG GBM xenograft resection model was used [32]. As previously mentioned, U87-MG cells transduced with optical reporters mCh-FLuc were stereotactically implanted into the brain parenchyma of mice (Figure 6A,B). Established tumors were surgically resected using fluorescence image guidance seven days later (Figure 6B and Supplementary Figure S5). Using the aforementioned dose (2.5 × 10^4^ iNSCs/mouse), iNSC-sTR were administered into the resection cavity (5 µL injection/mouse; n = 10) at equivalent dose via direct injection in sterile 1X DPBS or seeded in the CS hydrogel. The sham (resection without treatment) and iNSC-GFP-NLuc served as controls (n = 5/group). Tumor volumes were assessed using serial BLI to determine the overall survival and the mice were monitored three times weekly to observe changes in body weight and signs of morbidity (Figure 7A and Supplementary Figure S6). Animals that displayed clinical features of poor prognosis, such as weight loss > 20%, body condition score < 2, seizures, tumor growth > 1.5 cm, or complications following surgery during the course of the study were euthanized. Results showed that all mice experienced GBM recurrence in situ. It should be noted that one animal had died following surgery in the iNSC-sTR (direct injection) group due to surgical complications. The tumor volume in the iNSC-sTR treated animals was significantly smaller than the control treated animals. Furthermore, the tumors in the mice in the iNSC-sTR-CS hydrogel group were significantly smaller during the first 11 days post-treatment in comparison to the iNSC-sTR alone group (*p* < 0.05; Figure 7B). iNSC-sTR-CS hydrogel therapy also extended median survival by >50%, with the iNSC-sTR-CS hydrogel-treated animals surviving an average of 31 days compared to 15 days in iNSC-sTR-treated animals (*p* < 0.001; Figure 7C).

## 4. Discussion

There are more than 10,000 individuals that succumb to GBM annually in the US [33]. Tumoricidal stem cells (tSCs) have shown great promise as an emerging strategy to suppress the growth of GBM in preclinical and clinical studies. There have been challenges with its clinical use for post-surgical GBM due to the accelerated clearance from the resection cavity. Therefore, developing strategies to retain tSCs at the resection site can further improve anti-cancer treatment outcomes and prevent GBM recurrence. Chitosan-based hydrogels have been extensively used for regenerative medicine, drug delivery, and cell delivery applications [25,27,34,35,36]. Furthermore, CS is biocompatible, biodegradable, thermosensitive, and has antimicrobial properties. Here, we explore the biological properties of CS to create a novel injectable hydrogel to improve the clinical utility of iNSCs for post-surgical GBM treatment.

Previously, we determined that our CS hydrogel technology was permissive for cell encapsulation and maintained tumor killing functionality [27]. In the present study, we discovered that the CS hydrogel enhanced the initial retention of iNSCs and markedly prolonged their persistence in the resection cavity. Additionally, H&E analysis revealed that there were no delayed local-hypersensitivity events following implantation of the CS hydrogel. Moreover, results showed that tumoricidal iNSC-laden CS hydrogel therapy markedly reduced GBM tumor volumes in vivo and extended the median overall survival of post-surgical GBM bearing animals with more than a 50% increase in median survival relative to direct injection. Collectively, these findings suggest that utilizing an FDA-approved, injectable CS biomaterial to deliver iNSCs could be a promising new therapeutic approach to advance stem cell treatment for post-surgical GBM.

The ideal hydrogel scaffold for delivering stem cells (SCs) for GBM and other brain malignancies have not been fully elucidated. Ideally, the hydrogel’s gelation kinetics should be quick to reduce surgical time and maximize the retention of SCs in the resection cavity following implantation. The polymers should be biocompatible with the host, in situ forming to fit any resection cavity shape and/or size, and have no negative influence on cell phenotype, behavior, and functionality post encapsulation. Additionally, the hydrogel should mimic the mechanical and structural properties of the brain tissue to reduce inflammation and the need for removal following implantation. More importantly, the hydrogel scaffold should maximize stem cell survival, migration, cell-drug/protein release, and extend overall efficacy outcomes for GBM patients, given the inevitability of recurrence. The findings in this study demonstrated that the CS hydrogel significantly prolonged persistence of iNSCs from 21 days to up to 196 days, thus increasing persistence by up to 9.3-fold. Furthermore, the results demonstrated biocompatibility of the CS hydrogel with the murine host, suggesting ease of translatability with a human host. Because the CS hydrogel is in situ forming, we can utilize this injectable biomaterial with any resection shape or depth.

Novel treatment strategies for GBM are being developed. However, the efficacy of targeted therapies remain limited due to GBM heterogeneity and an inability to thoroughly target GBM receptors. Immunotherapy is another emerging approach for the treatment of GBM. However, GBM exhibits significant immune evasion, thereby minimizing its efficacy. With this novel approach, therapeutic iNSCs embedded in the CS hydrogel can be delivered once at the time of surgery, migrate to invasive tumor foci, and constitutively secrete TRAIL or other cytotoxic agents to completely eradicate GBM. Interestingly, our injectable CS hydrogel has the capabilities of being used as a bioink for 3D bioprinted use [37], therefore there is potential to create personalized, customizable hydrogel scaffolds for GBM patients, if necessary. This approach, similar to Gliadel, can (1) offer a higher delivery of cell payload at the target site, (2) minimize additional prepping steps at the point of surgery thereby minimizing surgical time, and (3) offer an easier method to clinicians for administering the therapeutic payload. As previously mentioned, there are several characteristics of the CS hydrogel that make it an ideal hydrogel scaffold for post-surgical GBM treatment; however, further studies are warranted to investigate the material’s influence on cell fate, behavior, phenotypic expression, and how this can impact anti-GBM killing functionality in vivo.

In this study, the CS-based hydrogel scaffold was able to reduce GBM tumor volumes in a post-surgical GBM mouse model. More interestingly, the iNSC-sTR CS-based hydrogel scaffold markedly extended the overall median survival from 15 days to 31 days, relative to iNSC-sTR direct injection. Notably, at days 7, 11, and 14 tumor radiance was significantly smaller in mice treated with the CS-iNSC-sTR compared to mice treated with a direct injection of iNSC-sTR as shown in the BLI images (Figure 7B). Tumor radiance for each mouse in each group was normalized to their BLI signals collected at day 1 post tumor resection. At day 21, although tumors were overall smaller in the CS-iNSC-sTR compared to iNSC-sTR direct injection, this difference was not significant. However, it is worth noting that at day 21, the number of mice in the iNSC-sTR group (n = 3; ~33% survival) was significantly lower than that in the CS-NSC-sTR group (n = 10, 100% survival), which could contribute to the loss in significance in tumor size between the two groups. Despite the significant reduction in tumor growth with iNSC-sTR delivered in the CS scaffold and as a result a significant increase in median survival (>50%) compared to direct injection of iNSC-sTR, tumors did relapse in all mice as demonstrated by the increase in tumor radiance (Figure 7B) and the Kaplan–Meier survival curve (Figure 7C). The mechanisms driving recurrence remains unclear and the need to further investigate the sensitivity of tumor cells following exposure to TRAIL secreting iNSCs postmortem is warranted. Additionally, the cell loading efficiency (dose) is low and further scaffold development strategies (e.g., CS molecular weight and percent composition) will be investigated to increase the cell density in the hydrogel scaffold and improve tumor efficacy and animal survival. Pertaining to this effort and to improve tumor killing, the CS hydrogel scaffold must be capable of retaining a therapeutic dose of iNSCs, prevent surgery-induced clearance of iNSCs, and permit cell release and migration to suppress GBM growth and prevent recurrence.

Undeniably, there are limitations in this study. There is extensive literature investigating the relationship between the polymer matrix and cells. It is known that physical and mechanical properties of biomaterials have an impact on cell characteristics and phenotypic changes while in the matrix. This work lacks studies aimed at evaluating changes in iNSC gene expression while encapsulated in our CS hydrogels. These changes can affect TRAIL secretion by downregulating the TRAIL gene, affect genes that regulate migration such as CXCR4, iNSC proliferation, and the differentiation of iNSCs back to fibroblast-like phenotype following encapsulation. As shown in Figure 4B and Figure 5C, iNSCs appear to proliferate in vivo according to the increase in BLI signal. However, postmortem analysis was not conducted to elucidate these findings. Therefore, future studies will evaluate the impact of material–cell interactions to further understand changes in vitro and in vivo. Interestingly, iNSC-sTR in CS hydrogels markedly improved overall median survival in comparison to all groups in this study. However, one notable concern with the efficacy study is the variability in image-guidance resections. As mentioned previously, the resections are performed using a fluorescence signal from the tumors under a surgical microscope. There is variability in removing the majority of the tumor and detecting positive resection margins following surgery as illustrated in Figure 6B and Supplementary Figure S5. Furthermore, it is difficult to determine the presence of a tumor that has migrated distally from the resection cavity. These differences between animals can be shown in Figure 7A where four out of nine animals iNSC-sTR (direct injection) group and four out of ten animals in the CS-iNSC-sTR group did not achieve maximal surgical resection. This variability in resections highlights the limitations of the resection model.

Lastly, we investigated the efficacy of the CS hydrogel scaffold to deliver iNSC-sTR using U87 human xenografts. U87 tumor cells form semi-solid tumors in vivo and are highly sensitive to TRAIL therapy. Given the lack of efficacy of these iNSC-sTR cells against U87 tumors that was demonstrated by other matrix systems mentioned previously, we used this tumor line to perform initial proof-of-concept studies. However, to fully evaluate the translatability of this combinatory technology for clinical application, we need to model diffuse GBM disease as seen in the clinic. Human GBM8 are tumor cells that result in a diffuse tumor that invades both brain hemispheres and are more sensitive to TRAIL therapy than U87 tumor cells. Hence, future studies are underway to explore the iNSC-laden CS hydrogel technology using GBM8 xenografts. Lastly, to fully understand the scalability of this innovative, combinatory technology we need to utilize a large animal model to better predict outcomes for human patients.

It should be noted that this monotherapy approach will unlikely be successful in GBM. Our goal in this work was to elucidate the potential of this combinatory technology for treatment of GBM. Recently, there have been efforts focused on immunotherapy for the treatment of GBM. CAR-T cell therapy has shown progress as a strategy to suppress tumor growth in preclinical studies [38,39]. Ogunnaike et al. engineered CAR-T cells targeting B7-H3 for treatment of post-surgical GBM [40]. This study demonstrated that encapsulating B7-H3 CAR-T cells in fibrin matrices significantly delayed U87 tumor growth in comparison to the intracranial administration of B7-H3 CAR-T cells. In this study, we demonstrate a significant increase in median survival with the CS scaffold despite a marginal delay in tumor growth overall. Moreover, the versatility of the CS hydrogel can be extended to (1) other cell therapeutics such as CAR-T cells for future studies of local GBM treatment (2) systemic delivery of drug/cell carriers for peripheral organ tumors, and (3) used as a personalized 3D bioink construct for the local delivery of drugs/cell carriers for treatment of GBM.

## 5. Conclusions

In summary, this research demonstrates the impact of CS hydrogel on iNSC persistence and efficacy with a minimal toxicity profile. Our work showed that the encapsulation of iNSCs in the CS hydrogel exhibited the longest persistence which ultimately extended the median overall survival in mice. Together, these data provide a framework for future iNSC-CS hydrogel-optimization studies with the potential to advance this technology in large animal models and human clinical trials.

## 6. Patents

SRB is an inventor on a patent application related to this work filed by the University of North Carolina, Office of Technology Commercialization (UNC OTC) (PCT International Application PCT/US2019/034492). The authors declare no conflicts of interest.

## Figures and Tables

**Table 1:** Composition of injectable CS hydrogel formulation.

Formulation	CS (% *w*/*v*)	BGP (mM)	HEC (mg/mL)	iNSCs (Per mL Hydrogel)
1	2	100	0.5	0
2	2	100	0.5	5 × 10^6^

## Data Availability

The data presented in this study are available within the article and Supplementary Material or on request from the corresponding author.

## Supplementary Materials

The following supporting information can be downloaded at: https://www.mdpi.com/article/10.3390/pharmaceutics17010003/s1, Figure S1: In vivo persistence of all animals post-implantation of iNSCs via direct injection or seeded in CS hydrogels.; Figure S2: Histology of healthy, untreated mice brain tissue; Figure S3: Microscopic findings in coronal brain tissue.; Figure S4: Histology of brain tissue in CS-treated animals brain tissue.; Figure S5: Fluorescence optical imaging of animal brain tumors before and after resections.; Figure S6: Body weight changes in single animals.
