# Supplementary material for: Injectable Tumoricidal Neural Stem Cell-Laden Hydrogel for Treatment of Glioblastoma Multiforme—An In Vivo Safety, Persistence, and Efficacy Study
Source: Pharmaceutics. 2024 Dec 24;17(1):3. doi: 10.3390/pharmaceutics17010003 (PMC11768746; doi:10.3390/pharmaceutics17010003)
Supplement: Supplementary file 1 [file pharmaceutics-17-00003-s001.zip › pharmaceutics-3340150-supplementary.pdf]

Article

Injectable Tumoricidal Neural Stem Cell-Laden Hydrogel for Treatment of Glioblastoma Multiforme—An In Vivo Safety, Persistence, and Efficacy Study

Jasmine L. King [1], Alain Valdivia [2] Shawn D. Hingtgen [2] and S. Rahima Benhabbour [1,2,*]

[1] Joint Department of Biomedical Engineering, North Carolina State University and The University of North Carolina at Chapel Hill, Chapel Hill, NC 27599, USA; jasmine_king@med.unc.edu
[2] Division of Pharmacoengineering and Molecular Pharmaceutics, UNC Eshelman School of Pharmacy, University of North Carolina at Chapel Hill, Chapel Hill, NC 27599, USA; alain07@email.unc.edu (A.V.); hingtgen@email.unc.edu (S.D.H.)
* Correspondence: benhabs@email.unc.edu; Tel.: +1-919-843-6142

Academic Editor: Pietro Matricardi

Received: 13 November 2024
Revised: 6 December 2024
Accepted: 17 December 2024
Published: 24 December 2024

Citation: King, J.L.; Valdivia, A.; Hingtgen, S.D.; Benhabbour, S.R. Injectable Tumoricidal Neural Stem Cell-Laden Hydrogel for Treatment of Glioblastoma Multiforme—An In Vivo Safety, Persistence, and Efficacy Study. *Pharmaceutics* **2025**, *16*, x. https://doi.org/10.3390/xxxxx

Copyright: © 2024 by the authors. Submitted for possible open access publication under the terms and conditions of the Creative Commons Attribution (CC BY) license (https://creativecommons.org/licenses/by/4.0/).

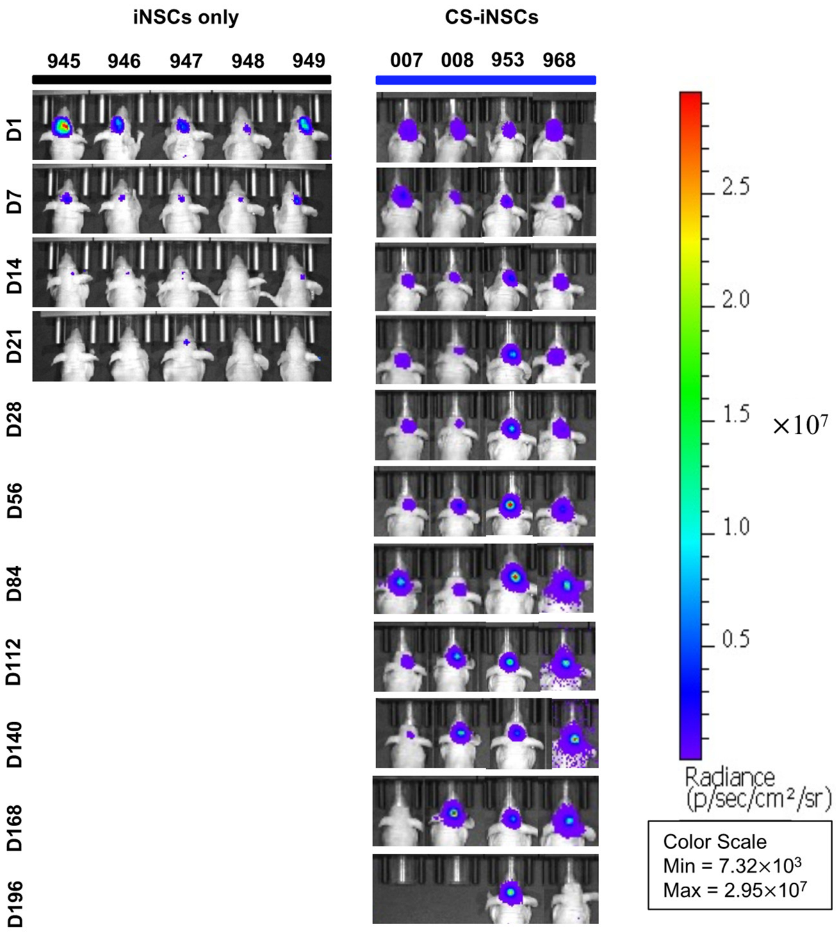

Figure S1. In vivo persistence of all animals post-implantation of iNSCs via direct injection or seeded in CS hydrogels.

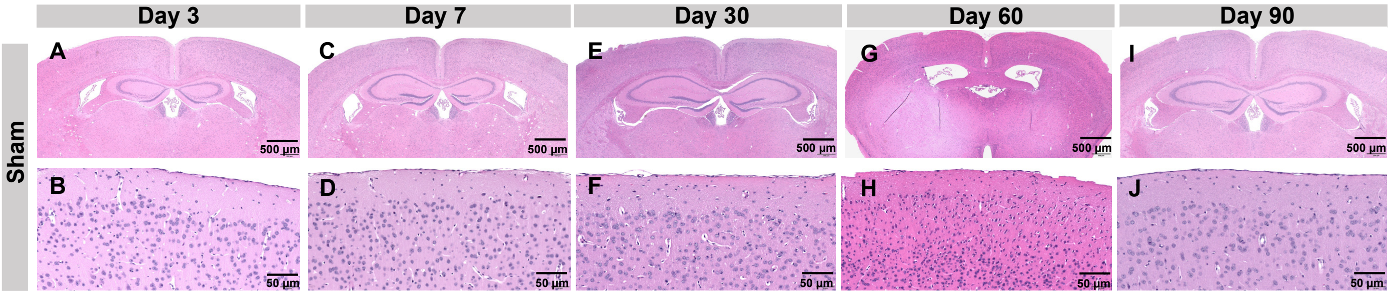

Figure S2. Histology of healthy, untreated mice brain tissue. Histological images of brain tissue at day 3 (**A-B**), 7 (**C-D**), 30 (**E-F**), 60 (**G-H**), and 90 (**I-J**). All scale bars (**A, C, E, G, I**) represent 500 µm. All scale bars for zoomed-in images (**B, D, F, H, J**) represent 50 µm. .

	Day 3			Day 7			Day 30			Day 60			Day 90		
**Microscopic findings in coronal brain samples**	**SHAM**	**Resection Control**	**CS Hydrogel**	**SHAM**	**Resection Control**	**CS Hydrogel**	**SHAM**	**Resection Control**	**CS Hydrogel**	**SHAM**	**Resection Control**	**CS Hydrogel**	**SHAM**	**Resection Control**	**CS Hydrogel**
**Resection site, unilateral (surgical site)**															
present	0	3	5	0	3	4	0	3	4	0	2	5	0	2	4
**Mixed inflammation, fibrin, mineral, resection site**															
present	NA	0	1	NA	0	1	NA	0	0	NA	0	0	NA	0	0
**Hemorrhage, parenchyma**															
minimal	0	2	2	0	1	0	0	0	0	0	0	0	0	0	0
mild	0	0	1	0	0	0	0	0	0	0	0	0	0	0	0
moderate	0	0	2	0	0	0	0	0	0	0	0	0	0	0	0
**Hemorrhage, unilateral, lateral ventricles**															
minimal	0	1	1	0	0	0	0	0	0	0	0	0	0	0	0
mild	0	0	0	0	0	0	0	0	0	0	0	0	0	0	0
moderate	0	1	1	0	0	0	0	0	0	0	0	0	0	0	0
**Necrosis/apoptosis, hippocampus**															
present	0	2	3	0	1	0	0	0	0	0	0	0	0	0	0
**Necrosis, neuronal, peri-resection**															
minimal	NA	1	0	NA	1	0	NA	0	0	NA	0	0	NA	0	0
**Gliosis, peri-resection**															
minimal	NA	1	0	NA	2	4	NA	3	4	NA	1	3	NA	2	3
mild	NA	0	0	NA	0	0	NA	0	0	NA	0	1	NA	0	1
**Swollen axons, peri-resection parenchyma**															
minimal	NA	1	0	NA	2	3	NA	0	2	NA	0	0	NA	0	0
**Edema/rarifaction, peri-resection**															
minimal	NA	3	1	NA	2	4	NA	2	4	NA	0	3	NA	0	3
mild	NA	0	4	NA	0	0	NA	1	0	NA	0	2	NA	0	0
**Infiltrates, macrophages, at surgical site**															
minimal	NA	1	1	NA	1	2	NA	0	1	NA	0	0	NA	0	0
mild	NA	2	4	NA	1	2	NA	0	0	NA	0	0	NA	0	0
**Pigmented macrophages at surgical site**															
minimal	NA	0	0	NA	2	3	NA	2	3	NA	2	3	NA	0	3
mild	NA	0	0	NA	0	0	NA	1	1	NA	0	2	NA	0	1
**Infiltrates, neutrophils, peri-resection**															
minimal	NA	2	3	NA	2	3	NA	0	0	NA	0	0	NA	0	0
mild	NA	0	1	NA	0	1	NA	0	0	NA	0	0	NA	0	0
**Fibrosis, hemorrhage, necrosis, bone spicules (fragments of calvaria from drilling)**															
present	NA	0	2	NA	1	1	NA	0	1	NA	0	0	NA	0	0
**Malacia/necrosis, hippocampal**															
mild	NA	0	1	NA	0	0	NA	0	0	NA	0	0	NA	0	0

Figure S3. Microscopic findings in coronal brain tissue. The number indicates animals from healthy untreated, resection-treated, and CS hydrogel-treated groups with listed microscopic findings.

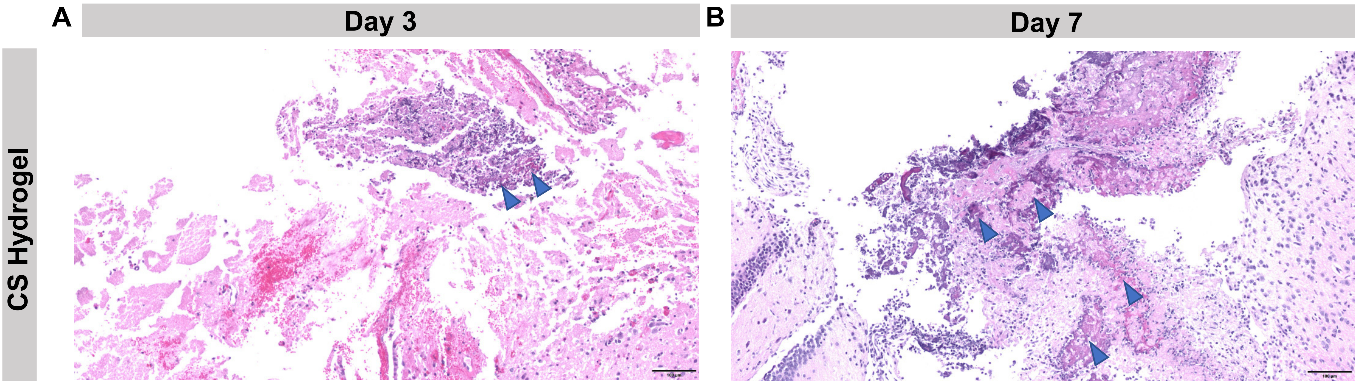

Figure S4. Histology images of brain tissue in CS-treated animals brain tissue. Mixed inflammation, fibrin, and mineral (indicated by blue triangles) identified in the resection site at day 3 (**A**) and day 7 (**B**). All scale bars represent 100 µm.

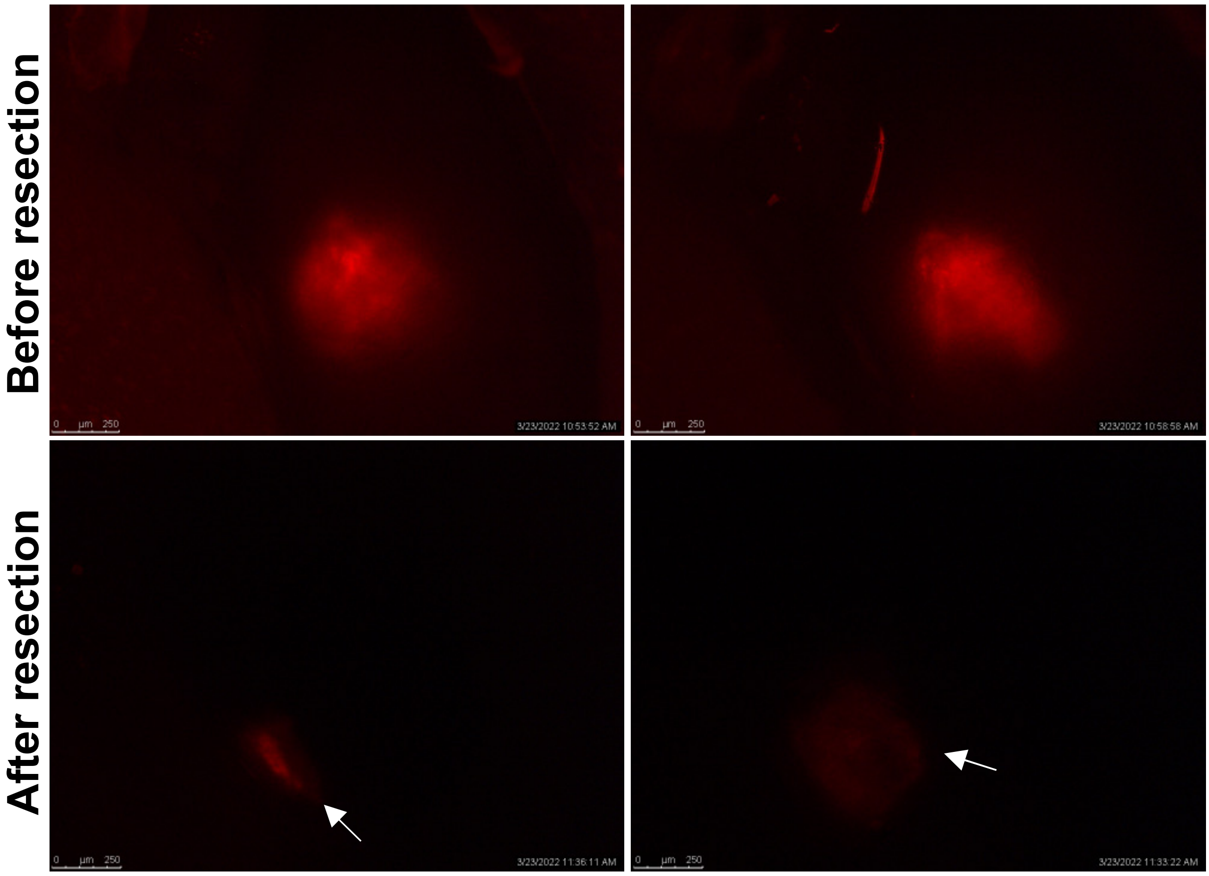

Figure S5. Fluorescence optical imaging of animal brain tumors before and after resections. White arrows show positive tumor margins following resections to mimic clinical scenario.

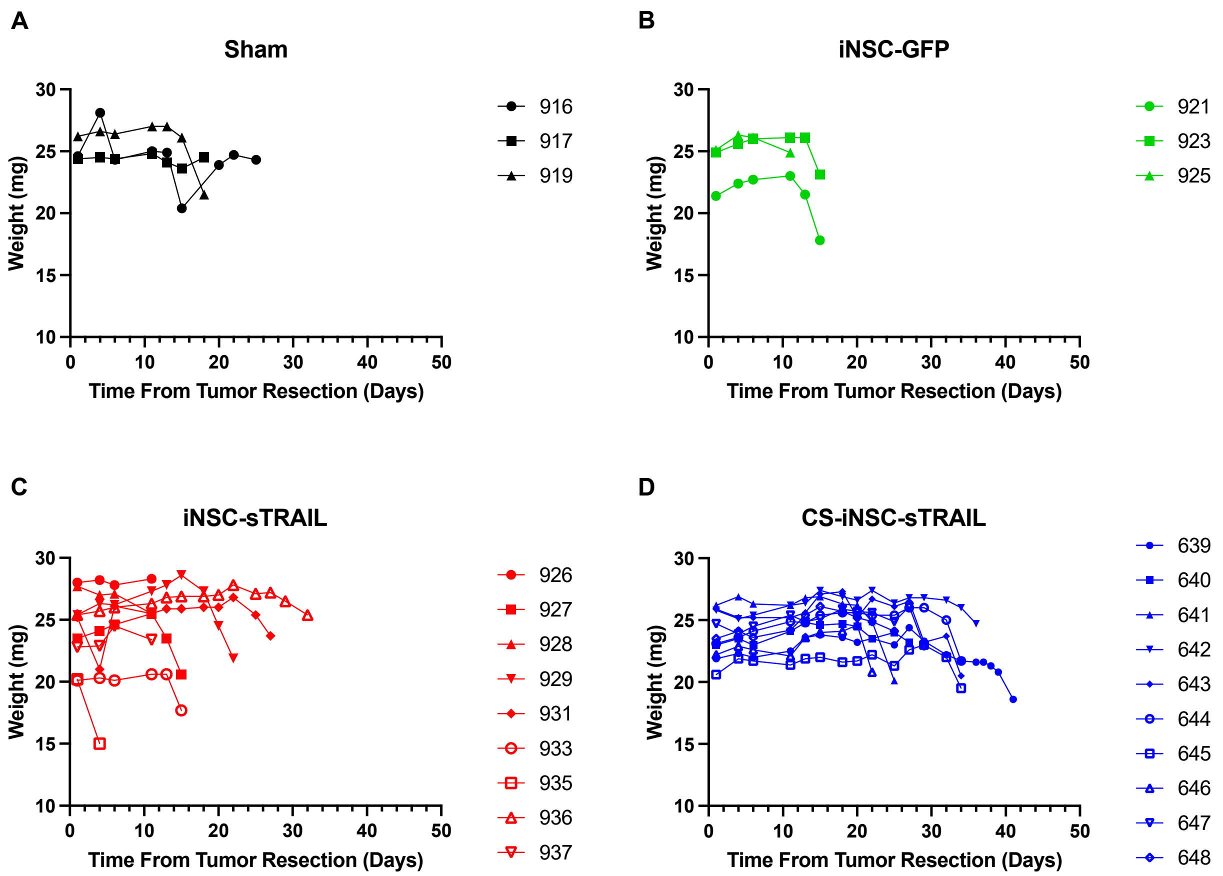

Figure S6. Body weight changes in single animals. Body weight was measured three times a week. Graph indicates body weight of animals in non-treated and treated-control **A**) sham and **B**) iNSC-GFP and treated groups **C**) iNSC-sTRAIL and **D**) CS-iNSC-sTRAIL, respectively.
